# Advancing health equity through CTSA programs: Opportunities for interaction between health equity, dissemination and implementation, and translational science

**DOI:** 10.1017/cts.2020.10

**Published:** 2020-01-28

**Authors:** Reza Yousefi Nooraie, Bethany M. Kwan, Elizabeth Cohn, Mona AuYoung, Megan Clarke Roberts, Prajakta Adsul, Rachel C. Shelton

**Affiliations:** 1 Department of Public Health Sciences, University of Rochester, Rochester, NY, USA; 2 Department of Family Medicine, School of Medicine, University of Colorado Anschutz Medical Campus, Aurora, CO, USA; 3 The Colorado Clinical & Translational Sciences Institute, Aurora, CO, USA; 4 Hunter-Bellevue School of Nursing, City University of New York, New York, NY, USA; 5 Scripps Whittier Diabetes Institute, Scripps Health, San Diego, CA, USA; 6 Division of Pharmaceutical Outcomes and Policy, UNC Eshelman School of Pharmacy, Chapel Hill, NC, USA; 7 Implementation Science Team, Division of Cancer Control and Population Science, National Cancer Institute, Rockville, MD, USA; 8 Department of Medicine, University of New Mexico, Albuquerque, NM, USA; 9 Department of Sociomedical Sciences, Mailman School of Public Health, Columbia University, New York, NY, USA

**Keywords:** Dissemination and implementation, health equity, translational science, CTSA, D&I

## Abstract

Dissemination and implementation (D&I) science is dedicated to studying how to effectively translate and apply research in real-world contexts. There has been increasing interest in health equity within the D&I field to ensure the equitable implementation of evidence-based programs/practices across a range of diverse populations and settings. At the same time, health equity researchers recognize the potential of D&I science to promote the more widespread dissemination, implementation, and sustainment of evidence-based interventions to address health inequities. The National Center for Accelerating Clinical and Translational Science Clinical and Translational Science Award (CTSA) Program has been a champion for community engagement and translational scholarship in its mission to improve individual and population health. The overall CTSA infrastructure and resources within and among CTSA hubs are well-equipped to facilitate a health equity focus to D&I across the phases of translational research. This paper proposes a framework that demonstrates the interaction and opportunities between health equity and D&I science and highlights how CTSAs can support and facilitate wider efforts in translational research with a focus on equitable D&I.

## Introduction

Dissemination and implementation (D&I) science is a dynamically growing field that focuses on reducing the gap between research and practice [1]. D&I science has established methods, strategies, and frameworks for facilitating the adoption, implementation, and ultimately sustainability of evidence-based interventions, guidelines, and programs (referred to here as EBIs) [1]. Aligned with recent conceptualizations of “equitable implementation” and health equity [2], EBIs will only have impact on the population health level if they are delivered equitably over time across diverse settings and populations.

There has been a growing recognition of the importance of health equity within D&I science [3, 4]. Health equity refers to providing a fair and just opportunity to be healthy, by “reducing and ultimately eliminating disparities in health and its determinants that adversely effect excluded or marginalized groups [5].” While the complex and embedded nature of context is reflected in the implementation frameworks like *The Consolidated Framework for Implementation Research* (CFIR [6]), *integrated-Promoting Action on Research Implementation in Health Services* (i-PARIHS [7]), *Exploration, Preparation, Implementation, Sustainment* (EPIS [8]), and *RE-AIM/PRISM* [9], these frameworks were not originally designed to explicitly focus on addressing health inequities.

Addressing equity has typically been an implicit objective of many approaches used within D&I (e.g., consideration of whether implementation of an EBI will be successful in low-resource settings or among populations that face more structural barriers) [10]. Additionally, health equity is sometimes an important driving force in the selection of settings or populations within D&I studies, particularly if that setting/population differs from that where the intervention was originally tested (e.g., D&I studies with a focus on addressing gaps in rural cancer clinics or addressing inequities across diverse racial or ethnic groups) [11]. Though D&I strategies have been applied to populations experiencing health inequities, there has been little consideration as to whether certain D&I strategies might be more appropriate or effective in addressing equity than others. Studies focused on cultural or contextual adaptation are also related to an interest in promoting equity or reducing disparities within D&I science [12, 13]. Notably, Baumann et al. proposed strategies to improve dialogue between D&I and cultural adaptation models, including incorporating a cultural adaptation lens to sensitize D&I planning and strategies, training and capacity needs for D&I research and practice, and applying participatory approaches to engage diverse stakeholders [14]. Recent work by McNulty et al. and Chinman et al. has also provided examples of how to incorporate a focus on health disparities and health equity into implementation science study design [15, 16].

There are many reasons why health equity has been more implicit or exploratory in the context of D&I, including some of the methodological, funding, and resource challenges (e.g., needing large and diverse samples to examine inequities across settings or populations), as well as structural barriers that have limited historically marginalized populations from participating in research (which are still being addressed to this day, e.g., medical mistrust). Despite these challenges, we believe there is value and potential impact in making health equity an explicit part of D&I models/frameworks, measures, and planning, execution, and evaluation processes.

In considering opportunities to systematically bring an equity lens to D&I science, we assert that there are existing opportunities to leverage the resources, infrastructure, and expertise of Clinical and Translational Science Awards (CTSAs). D&I and translational science (the latter as reflected in CTSAs) are well-aligned given their common goals of improving the dynamic processes along the translational continuum that contribute to the well-documented gap between research and practice [17]. There has been growing recognition of opportunities to bridge between translational science (that is concerned with the problem-oriented translation of observations in the laboratory, clinic, and community into interventions to improve the health of individuals and the public) and D&I [17, 18]. We also assert that CTSAs may be well suited to provide a fitting infrastructure and setting to bring greater focus on health equity and D&I.

CTSAs provide major translational research infrastructure in ~60 academic medical research centers (“hubs”) across the USA and are strategically positioned to help advance D&I research, practice, and training nationally. These CTSA hubs are critical in fostering collaboration between multidisciplinary investigators to: (1) facilitate innovative translational research and training across all stages of translational continuum; (2) provide training to support workforce development; and (3) to develop, demonstrate, and disseminate effective research tools and solutions to overcome translational roadblocks [19].

Recognition of how health equity, D&I, and translational science intersect and how these domains can inform and sensitize one another and be operationalized can facilitate the development of more integrated conceptual frameworks, and ultimately the development, dissemination, implementation, and sustainability of effective interventions to address inequities and bridge knowledge-to-action gaps [15]. This paper is organized in two sections: (1) a proposed framework (*the EQ-DI framework*) that highlights the interaction opportunities between D&I and health equity; and (2) suggested solutions and examples of how CTSAs can provide key support, resources, and infrastructure to promote D&I, with a more explicit focus on health equity in translational research.

## EQ-DI Framework: Interaction Between Health Equity and D&I

There are many commonalities and synergies among the two action-oriented science fields of D&I and health equity. For example, both recognize the multi-level and embedded nature of context and the need for adaptation to the unique needs and characteristics of key populations, settings, and stakeholders. Additionally, both approaches value stakeholder engagement, and have long-lasting ties with community-based participatory research (CBPR) [20, 21], as reflected in the Transcreation Framework [4].

There are many areas within D&I science that can be enhanced by scholarship on health equity. As one example, the specific aspects of context that are most relevant to health inequities (e.g., social determinants of health, discrimination and other forms of structural and inter-personal racism, and medical mistrust), and considerations of intersectionality (i.e., understanding and addressing health inequities through recognizing mutual influence of various dimensions of social and political life [22]) have not been well-represented in D&I science. D&I strategies may actually exacerbate health inequities if they are not consciously recognized and addressed [23]. Examples include the diffusion and dissemination of health innovations through personal digital platforms (e.g., patient portals) and social media communities, which require reliable Internet access (e.g., smartphones with data plans and broadband internet) and language proficiency, potentially deepening the digital divide for populations with limited access to technology/internet. These digital tools are increasingly being used in the pursuit of precision medicine, intending to provide more tailored treatments to individuals, which could lead to potential exacerbation of health inequities if an explicit health equity lens is not built into their D&I plans.

**Fig. 1.**
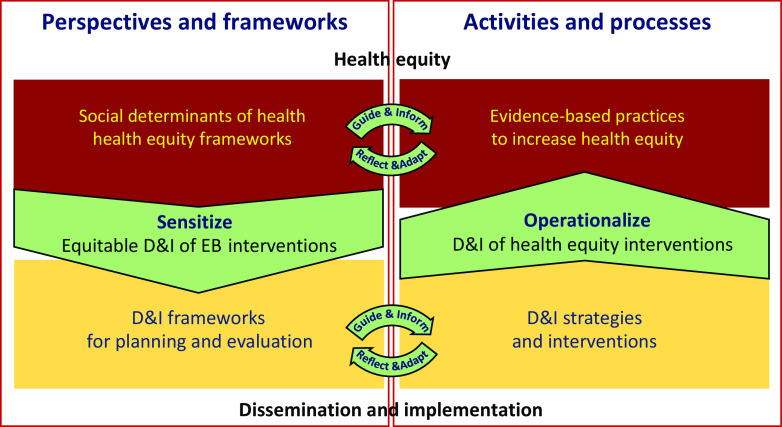
EQ-DI framework on the interaction between health equity and D&I.

Building off of existing health equity and D&I scholarship and literature, we propose that two central domains of health equity and D&I interact, as depicted in the *EQ-DI framework* (Fig. 1). Health equity concepts can sensitize D&I planning and evaluation frameworks to consider multi-level and complex socio-ecological dynamics that may effect equitable D&I (Fig. 1; left half). On the other hand, D&I science can help operationalize the D&I of evidence-based interventions proposed to promote health equity, by providing toolkits, methodologies, approaches, and evaluation plans that make equity central (Fig. 1; right half).

### Sensitizing

Health equity could be a lens to sensitize and inform D&I planning, execution, and evaluation. Below, we outline ways in which D&I could be enhanced by a health equity “sensitizing” lens.

A D&I approach that is sensitive to health equity recognizes how inequities are created, addressed, and reinforced through complex interactions within and across multiple socio-ecological levels of health. A social–ecological perspective to D&I will acknowledge and plan for the embeddedness of individuals in relational, community, and system-level contexts, and the need for multi-level interventions and implementation strategies to address the complexity of health inequities [24].

A sensitized D&I approach also recognizes the socially bounded nature of diffusion, dissemination, and implementation, and the need for active strategies to reach specific communities, settings, and sub-populations. For example, the *ConNECT* Framework has been developed to incorporate a health equity lens across the translational research continuum, from discovery to dissemination, by providing five actionable principles: integrating CONtext (by addressing social and contextual determinants of disparities), fostering a Norm of inclusion (by maximizing diversity of research participants), ensuring Equitable diffusion of innovations (by engaging diverse users for dissemination, participatory approaches to engage stakeholders from conceptualization to dissemination), harnessing Communication technology (leveraging smartphone and internet technologies to reach hard to reach populations), and prioritizing specialized Training (mentoring disadvantaged investigators, transcultural relevance of the trainings, health equity as a guiding principle of education, and workforce development for healthcare professionals and researchers)[25, 26].

A sensitized framework to D&I will explicitly consider health equity as an important aspect of the adaptation of EBIs to local contexts and populations prior to implementation. For example, the *Framework for Reporting Adaptations and Modifications-Enhanced* has been refined [27] to provide more context around cultural adaptations and modifications to EBIs, including who was involved in making the decisions for modifications (e.g., community members and political leaders) and the reasons for them.

Explicit evaluation of implementation from an equity lens could identify and track inadvertent deepening of health inequities, as in cases where populations and organizations with more resources or fewer structural barriers are more able to implement new innovations. There is a need for more D&I frameworks and evaluation approaches that explicitly capture this. For example, Glasgow et al.’s application of the *RE-AIM Framework* to plan and evaluate the implementation of a weight loss and self-management intervention with an emphasis on health inequities in all phases of reach (aiming to access disparate populations), effectiveness (e.g., assessing patient-centered outcomes and the effect of context), adoption (documenting and enhancing participation of low-resource setting), implementation (monitoring the delivery to diverse groups), and maintenance (assessing long-term impact among diverse groups) is an example of sensitizing an implementation framework to plan for and evaluate the process through the lens of health equity [11]. As another example, Woodward et al. [28] applied a health equity lens to sensitize i-PARIHS implementation framework and used it to guide qualitative interviews among Black patients on how health equity comes into play at various aspects of the uptake of hepatitis C treatments.

**Fig. 2.**
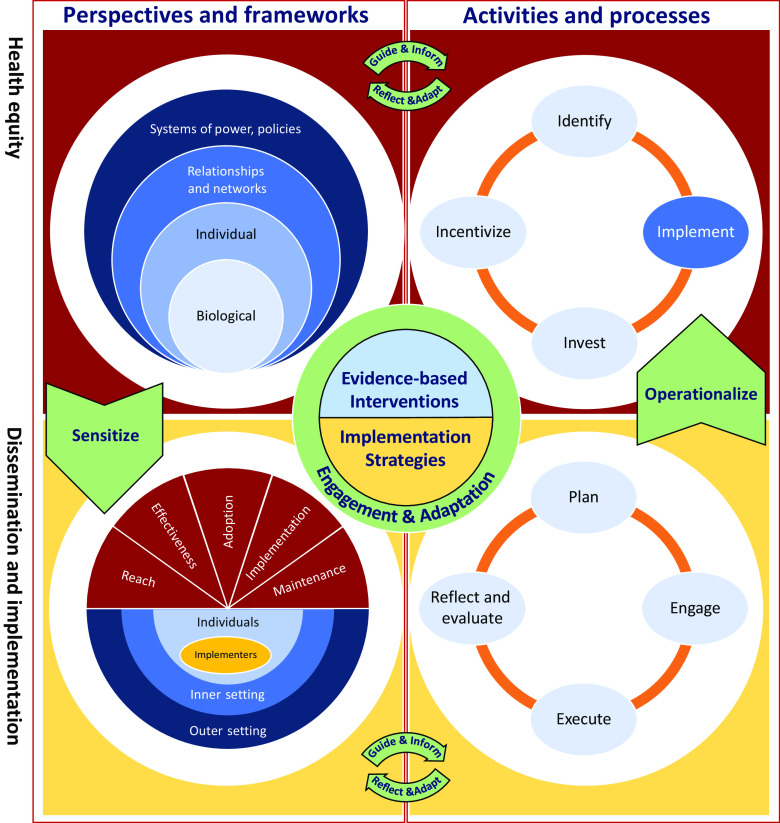
Extended representation of EQ-DI framework of the interaction between health equity and D&I.

Fig. 2 shows an example of possible expansions of the *EQ-DI framework* in which we used some domain-specific models to fill in the quadrants. The top left quadrant presents a social–ecological perspective to understanding social determinants of health and health equity, which sensitizes D&I planning and evaluation (lower left quadrant) by recognizing the embedded, contextual, and multi-level nature of health systems and communities (adapted from CFIR [6]). A sensitized D&I evaluation framework considers health equity at various phases of reach, effectiveness, adoption, implementation, and maintenance (informed by RE-AIM [9]).

### Operationalizing

On the other hand, D&I can provide a foundation for and guidance on how to operationalize the dissemination, implementation, and impact evaluation of EBIs to address health inequities. Over the years, several personal-, inter-personal-, and structural-level interventions have been developed and tested to address health disparities, and there is a widespread call for innovative EBIs. Examples of interventions at personal and inter-personal levels include exposure to counter-stereotypical exemplars to overcome implicit biases in health professionals [29], and stigma reduction and management [30, 31], which have shown some evidence of effectiveness. Structural interventions that aim to change the physical and built environments (such as housing mobility interventions [32]), sociocultural determinants, and multiple levels of influences [33] are complex and more difficult to assess [34] but still show promise in addressing health inequities. As interest in and evidence for such interventions grows, more robust planning and evaluation of sustainability, scalability, and replicability of successful interventions are needed [34].

Fig. 2 provides an expanded example of the *EQ-DI framework* and the role of D&I in operationalizing health equity research, where health equity interventions are positioned as the product of the roadmap for promoting health equity, using the roadmap developed by the National Quality Forum [35] (upper-right quadrant). The roadmap emphasizes the role of implementation as a main component, which is operationalized using D&I knowledge-to-action processes [36] (lower right quadrant). At the center of the figure, interventions (evidence-based innovations, practices, programs to increase equity, as well as strategies to help disseminate and implement them) are the result of intersections of the four quadrants and are developed and adapted through continuous engagement of stakeholders. While we recognize some of the challenges in rapid cycle D&I research and being able to act upon findings following reflection and evaluation processes, we assert that engagement of stakeholders is a key ingredient in all phases of this iterative, bi-directional process [4, 37].

## EQ-DI and Translational Research: The Role of CTSA Institutions

We have made the case that D&I and health equity complement each other through sensitizing D&I conceptualization and evaluation through a health equity lens, and operationalizing the D&I of evidence-based health equity interventions. We argue that CTSA programs have the potential to support EQ-DI by providing services and the infrastructure to facilitate the interaction between D&I, health equity, and translational science in part by leveraging existing priority areas of CTSAs including community and stakeholder engagement, workforce development and training, and team science, and providing financial support of priority areas across the translational research continuum.

### Community and Stakeholder Engagement

Optimal communication between health equity and D&I science requires active engagement of a broad range of stakeholders. Community engagement and co-production of knowledge are complex tasks requiring expertise, organizational support, and infrastructure to be successful and cost-effective [38]. CBPR lies on one continuum of engagement, with equitable sharing of power, resources, and decision-making throughout the research process [21]. CTSA hubs provided the infrastructure to improve community engagement [39] as they include Community Engagement Cores that help build capacity in communities and researchers to participate as full partners. The CTSA has a unique ability to lead in this area because in addition to D&I experts, the program also includes experts in community-engaged research and CBPR, as well as team science (learning how to move toward a transdisciplinary approach with stakeholders from multiple fields and backgrounds as described below). As the common thread in these areas, CTSA programs can facilitate the meaningful engagement of community and other stakeholders in D&I activities and research by prioritizing community engagement in funding and other support activities, offering training sessions on community-engaged research and CBPR, and providing awards for excellence in developing academic-community partnerships aimed at reducing health inequities.

### Workforce Development and Training

The past decade has seen the rise of multiple D&I training opportunities (including graduate programs, workshops, mentored research courses, and career development awards), in response to the widely recognized need to increase D&I workforce capacity [40]. Recent efforts have also focused on building D&I practitioner capacity through web-based education, technical assistance, self-directed learning, online toolkits, communities of practice, and multi-strategy interventions. Training and capacity building through mentorship programs is another effective strategy to build skill sets and potential research collaborations and networks [41]. The CTSA provides the infrastructure to integrate training and mentorship, tool development, professional support, and consultation for research proposals. The size and design of CTSA also lends itself to developing networks and connections within and across systems and institutions. Specifically, health equity and D&I could be incorporated into the curricula and topics of CTSA trainings (informal and through formal training/career development programs), at educational/workforce development meetings, through engagement of D&I and health equity experts in mentorship teams, and through the provision of methodological consultation or support to operationalize and guide the implementation of health equity-focused EBIs.

### Financial Support

Providing financial support to pilot studies, career development, interdisciplinary/team science, and early-stage translational endeavors has been an important aspect of CTSA program, including specific awards for D&I and/or community engaged research. CTSA institutions have used such resources to address priority topics and promote translational research collaborations across sites. CTSA programs can bring attention to the communication and dissemination opportunities between health equity and D&I by emphasizing health equity-sensitized D&I as a key outcome or focus in these awards. CTSA funding could be used to promote and support studies of dissemination, implementation, and sustainability of interventions addressing health inequities, and studies aiming to promote equitable D&I of EBIs. D&I approaches focused on health equity could also be leveraged in collaboration with other key resources or cores within CTSAs that are natural partners for a focus on D&I and health equity (e.g., Integrating Special Populations Resource within CTSAs).

## Summary, a Successful Example, and Future Directions

As practitioners and D&I researchers consider the inclusion of health equity in their work, the National Center for Accelerating Clinical and Translational Science (NCATS) CTSA program has the opportunity to integrate D&I principles across multiple aspects of translation (as part of its broader mission to accelerate the delivery of new treatments and cures for disease to patients). Aligned with this goal, we propose the additional incorporation of an explicit health equity perspective. NCATS continues to expand and leverage research teams that include scientists, providers, patient advocacy organizations, and community members to tackle system-wide scientific and operational challenges in clinical and translational research. This equity lens also aligns with recent CTSA programmatic goals (https://ncats.nih.gov/ctsa/about), including engaging patients and communities in every phase of the translational process, promoting the integration of special and underserved populations in translational research across the human lifespan, innovating processes to increase the quality and efficiency of translational research, and training and cultivating the translational science workforce.

Many CTSA institutions have pioneered activities toward these goals. As a successful example, the Colorado Clinical & Translational Sciences Institute (CCTSI) incorporated health equity, D&I, and translational science in its training and resource support services [42] (see Table 1):

- The CCTSI is especially strong in its support for building capacity in community engagement – noted as a foundational strategy for D&I and essential to implementation of EBIs for health equity. Investigators can learn principles and approaches to engagement, build relationships with community, and gather pilot data on research developed in collaboration with community. For instance, the Colorado Immersion Training program [43] has trained over 100 people in the principles of CBPR, health disparities, listening and self-reflection skills, and engagement tools. Participants receive directed readings, seminars, an immersion experience in a priority community (e.g., urban African American, refugee, urban Latino, and rural), and 6 months of faculty mentoring support.
- CCTSI members can apply for community engagement-specific funding opportunities to support partnership development with communities (e.g., prioritize research questions or operationalize implementation and study of interventions that address community needs) or to support pilot research from those partnerships.
- The CCTSI Partnership of Academicians and Communities for Translation Council provides oversight for all community engagement activities, with an eye toward reducing health disparities in Colorado.
- The Community Research Liaisons (CRLs) are trained partners from Colorado communities who support partnerships between researchers and individuals within a community. They are a highly valued resource for those conducting clinical and translational research and who are seeking to engage and partner with communities experiencing disparities. CRLs have supported a variety of projects that have been designed for D&I in collaboration with communities, including research on aging in place among lesbian, gay, bisexual, and transgender communities [43], and co-designing educational materials to support community engagement in big data research [44].
- As shown in the *EQ-DI framework*, understanding the needs, perspectives, strengths, and assets of communities is an important first step to sensitizing and informing interventions to address health equity. Then D&I models and frameworks can be applied to operationalize the implementation and testing of these interventions. The CCTSI D&I infrastructure and resources – in partnership with the University of Colorado’s Adult & Child Consortium for Health Outcomes Research and Delivery Science (ACCORDS) D&I program – provide training, education, consultation, and online tools to assist investigators with implementation (including adaptations) and evaluation of EBIs designed in collaboration with community. For example, a project that began with community and stakeholder engagement using the CCTSI’s Boot Camp Translation training led to the design of a comparative effectiveness study of diabetes group visits [45]. The Invested in Diabetes study, funded by the Patient-Centered Outcomes Research Institute in 2017, is now being tested in diverse settings (including low-resource federally qualified health centers, and practices with large Hispanic/Latinx and Native Health populations) using the enhanced Replicating Effective Programs (REP) implementation framework and the RE-AIM evaluation framework, with support from University of Colorado D&I experts. The Invested in Diabetes study operationalizes the implementation of equity EBIs using D&I frameworks in three key ways. First, research prioritization was informed by stakeholder needs and perspectives (i.e., foundation in stakeholder engagement) regarding patient health literacy and disease-related distress in the context of diabetes self-management support in primary care. Second, the enhanced REP D&I framework was used to adapt and implement an EBI (the Targeted Training in Illness Management program) for delivery in contexts with known health disparities: low-resource primary care settings, people with co-occurring mental health conditions, and people who speak Spanish. REP explicitly includes pre-implementation stakeholder engagement in packaging the EBI for delivery in specific clinical environments. Third, the RE-AIM framework encourages consideration of reach and effectiveness of interventions in the target populations and settings. The Invested in Diabetes study will test *a priori* hypotheses for subgroup analyses of group participation and outcomes in federally qualified health centers vs. commercial payer practices, patients with and without co-occurring mental health conditions, and those participating in Spanish vs. English group visits.

**Table 1.** Opportunities at CTSAs to facilitate interactions between health equity and D&I

EQ-DI framework	Implications for CTSAs	Example of Colorado Clinical & Translational Sciences Institute (CCTSI)	
		CCTSI activities	CCTSI guiding principles
*Sensitizing* D&I: Understand and target the embedded and intersectional nature of determinants of health and disparities	Community and stakeholder engagement Workforce development and training Channeling financial resources and funding supports	Colorado Immersion Training in community engagement Research studios D&I consults Community consults Community Research Liaisons Community engagement partnership development and joint pilot grants	Community-based participatory research principles: acknowledging centrality of the community perspective, empowerment and power sharing processes with attention to social inequalities, co-learning and capacity building, focus on multiple levels of determinants of health, long-term commitment to relationships and sustainability
*Operationalizing* the dissemination, implementation, and sustainability of health equity EBIs	Community and stakeholder engagement Workforce development and training Channeling financial resources and funding supports	Workshops, seminars, courses, and online tools in D&I methods, measures, and models Consultation and support for “designing for D&I” Collaborative team science with expertise in multiple disciplines and quantitative, qualitative, and mixed methods research Practice-based research networks Health policy and system partnerships	Pragmatic research principles: understanding, assessing, and designing for diverse clinical and community contexts and populations using multiple methods, inclusive participation criteria, and patient-centered outcomes measures Multi-level and multi-system perspectives: diverse research settings and data sources to assess and promote generalizability and sustainability

## Conclusions

In order to facilitate and sustain such efforts and track progress toward health equity, there is a need for guiding frameworks and actionable strategies. In this paper, we argued that CTSA hubs are well-equipped to enhance and leverage the interaction between D&I and health equity. We suggest opportunities for this integration through the following (Table 1):

- Incorporating D&I and health equity into goal setting, mission development, and training of transdisciplinary teams. Even within institutions, CTSA cores are not always well-integrated and can be siloed at times. The cross-disciplinary nature of EQ-DI provides opportunities to align various cores and functions (e.g., stakeholder engagement, workforce development and training, etc.) to achieve common goals.
- Providing D&I infrastructure and support to research teams to meaningfully engage community members and other key stakeholders in priority identification, planning, implementation, and evaluation; all of these components of engagement have critical implications for long-term sustainability of partnerships, programs, and health impact.
- Incorporating health equity-sensitized D&I into training, mentorship programs, professional support, toolkit development, and ongoing workforce development, including the diversification of workforce to reflect the communities served and ultimately help facilitate health equity efforts.
- Channeling more financial resources into D&I within CTSAs, including prioritizing health equity-sensitized EBIs and strategies, and operationalizing the dissemination, implementation, and sustainability of interventions to address health equity.
- Promoting communication and collaboration among researchers, knowledge users, and community members through network-building, and using the hub and spoke structure of CTSA programs as dissemination channels. Through this network, there are also opportunities to proactively test the most impactful dissemination strategies for reaching diverse audiences. Cross-CTSA collaboration can also help build capacity for both D&I and health equity, particularly in sites that do not currently have but would like to build this expertise.

These opportunities provide some examples of the extensive capabilities of CTSA programs in incorporating health equity and D&I across the translational spectrum. While we have focused on opportunities that lay later in the translational continuum (T3, T4), we encourage researchers to consider and engage through CTSAs to consider how equity may also be embedded earlier along the continuum as well. We recognize that there will be challenges in moving forward this agenda (e.g., variation across CTSAs in capacity to address D&I and equity, potentially limited financial support to facilitate such work with CTSAs, misperceptions in the scientific community as to what D&I research entails, and scientific cultural shifts needed to prioritize equity and community-engaged research). However, we believe that this integration is essential to achieve the broader goals of CTSA programs to ultimately advance research translation for both community and clinical partners, including learning health care systems [47].
